# Validity of Scottish predictors of child obesity (age 12) for risk screening in mid-childhood: a secondary analysis of prospective cohort study data—with sensitivity analyses for settings without various routinely collected predictor variables

**DOI:** 10.1038/s41366-022-01157-5

**Published:** 2022-06-03

**Authors:** Gabriela Carrillo-Balam, Lawrence Doi, Louise Marryat, Andrew James Williams, Paul Bradshaw, John Frank

**Affiliations:** 1 Usher Institute, University of Edinburgh, Edinburgh, UK; 2 School of Health in Social Science, University of Edinburgh, Edinburgh, UK; 3 School of Health Sciences, University of Dundee, Dundee, UK; 4 School of Medicine, University of St Andrews, St Andrews, UK; 5 Director, ScotCen Social Research, Edinburgh, UK

**Keywords:** Epidemiology, Obesity, Epidemiology, Disease prevention, Risk factors

## Abstract

**Objective:**

To analyse the Growing Up in Scotland cohort for predictors of obesity at age 12, present at school entry (age 5–6).

**Methods:**

The initial model included literature-based risk factors likely to be routinely collected in high-income countries (HICs), as well as “Adverse/Protective Childhood Experiences (ACEs/PCEs)”. Missing data were handled by Multiple Chained Equations. Variable-reduction was performed using multivariable logistic regression with backwards and forwards stepwise elimination, followed by internal validation by bootstrapping. Optimal sensitivity/specificity cut-offs for the most parsimonious and accurate models in two situations (optimum available data, and routinely available data in Scotland) were examined for their referral burden, and Positive and Negative Predictive Values.

**Results:**

Data for 2787 children with full outcome data (obesity prevalence 18.3% at age 12) were used to develop the models. The final “Optimum Data” model included six predictors of obesity: maternal body mass index, indoor smoking, equivalized income quintile, child’s sex, child’s BMI at age 5–6, and ACEs. After internal validation, the area under the receiver operating characteristic curve was 0.855 (95% CI 0.852–0.859). A cut-off based on Youden’s J statistic for the Optimum Data model yielded a specificity of 77.6% and sensitivity of 76.3%. 37.0% of screened children were “Total Screen Positives” (and thus would constitute the “referral burden”.) A “Scottish Data” model, without equivalized income quintile and ACEs as a predictor, and instead using Scottish Index of Multiple Deprivation quintile and “age at introduction of solid foods,” was slightly less sensitive (76.2%) but slightly more specific (79.2%), leading to a smaller referral burden (30.8%).

**Conclusion:**

Universally collected, machine readable and linkable data at age 5–6 predict reasonably well children who will be obese by age 12. However, the Scottish treatment system is unable to cope with the resultant referral burden and other criteria for screening would have to be met.

## Background and rationale

The global obesity pandemic is proceeding apace, with little evidence as yet that any jurisdiction has successfully controlled its growth, despite many well-intended intervention policies and programmes of diverse types [1–3]. Among these interventions, several studies have prospectively evaluated the use of universal or targeted screening, to predict child obesity before it becomes well established and relatively difficult to treat successfully [3]. A pre-requisite for such screening is a validated predictive algorithm based on universally available (routinely collected), machine-readable (i.e., amenable to computerised analysis to enable efficient population-based screening) and linkable predictor variables, derived from prospective cohort studies with high-quality predictor and outcome data – ideally population-based.

The volume and variety of studies developing (and in some cases validating, internally and/or externally) such predictive models have grown rapidly over the last decade, to the point where a number of systematic reviews have been published, identifying the primary studies’ respective strengths and weaknesses, and making recommendations for methodological improvement [4–6]. Almost none of the primary studies reviewed, however, have examined the comparative validity of prediction models in mid-childhood for later childhood obesity, especially pre-pubertally, when the prognosis for adult health is largely set in place, given that overweight children’s weight-for-height tracks strongly into adulthood [7]. No studies we are aware of have examined which routinely collected predictor variables, from a variety of routinely collected databases, are critical to the reasonably valid prediction of obesity pre-pubertally. This study, therefore, addresses the research question: Which predictors, collected between antenatal life and age 5–6, from what kinds of routinely collected databases, allow reasonably valid prediction of obesity at age 12?

This study analyses a high-quality child cohort, the Growing Up in Scotland Study (GUS), originally representative of infants born in 2004/5 and living in Scotland at 10 months of age, to derive a range of multivariable predictive models for obesity at age 12 (the latest follow-up wave on this cohort), using a range of predictor variables that are largely routinely collected, machine-readable and linkable in various High-Income Countries (HICs). The overarching goal is to identify the key data-elements for national child obesity surveillance systems in HICs, based on data collected universally in early to mid-childhood, for the prediction of obesity at age 12, as a first step towards universal screening at, for example, school entry (age 5–6 in Scotland). The aim of such screening would be the early identification of children at high risk, and the offering of effective child/family treatment before the child’s obesity is fully established – and almost certainly less treatable [3] – in pre-pubertal life.

## What is known about risk factors for childhood obesity in HICs?

A thorough systematic review [8] of 282 epidemiological studies in HICs assessed potential risk factors for childhood obesity, measured from prenatal life to age 2 years - since 0–2 years is the age range within which most routinely collected data on potential risk factors are available in most HICs. That review, combined with a very recent Dutch prognostic study [9], systematically identifying all “candidate predictor variables in the literature for predicting obesity at age 8,” found the following risk factors to be replicated across a number of high-quality studies (those in brackets have been replicated in far fewer studies and so are not as clearly evidenced):

- Male sex of child
- Various markers of parental socioeconomic status, including maternal education, family income, and deprivation-index of residential address – all routinely collected in Scotland
- Maternal pre-pregnancy data: high body mass index, (low parity)
- Maternal pregnancy data: high gestational weight gain, gestational diabetes, smoking
- Birth data: birthweight (Caesarean birth)
- Infancy and early childhood data: high weight gain in first year of life, no breastfeeding; (early feeding of solids); (low socioeconomic status); (low maternal bonding); (high antibiotic use); (childcare attendance)

Of these potential predictors of later childhood overweight/obesity, almost all (except the last three listed, within the last bullet) are routinely collected in many HICs. However, they have been as yet little utilised in national surveillance systems for monitoring or predicting childhood overweight/obesity, with a view to informing prevention.

Some of the candidate risk factors listed above require primary data collection after the perinatal period. In Scotland these data are collected during Health Visitor home visits, which, since 2013, have been routine up to age 27–30 months (recently changed to 4–5 years); data include duration of breastfeeding; age of introduction of solid foods, and presence of smoking in the home of the child [4–6, 8, 9]. Other predictor variables listed above may not be routinely collected in some countries – e.g., weight and height at age 5–6, but are collected in Scotland, in a universal Primary 1 (first grade) examination, which could easily provide the basis for a more comprehensive screening for a wider range of risk factors for obesity at a later age. Since this is the first age at which a very large proportion of each birth cohort are accessible for physical examination as they enter school, we have used body mass index (BMI) at age 5–6 in all our prediction models for obesity at age 12, rather than BMI at other ages.

In previous work with the GUS obesity data, we published [10] a multivariate predictive analysis identifying the following independent risk factors for having an “obesogenic growth trajectory” between ages 4 and 8:

- overweight/obesity in the mother during the child’s mid-childhood
- maternal smoking in the pregnancy.

In 2018, a detailed body-weight-for-heights analysis for GUS subjects followed up to age 10 showed rapid increases in the rates of obesity and overweight between ages 6 and 10, as well as widening inequality in these rates by various measures of social class (family income, as well as area deprivation of family residence measured via the Scottish Index of Multiple Deprivation [SIMD]) [11].

Two recent publications by our group have found surrogates within the GUS dataset for the majority of questions comprising the “Adverse Childhood Experiences (ACEs)” score [12–14]. Several studies have explored the relationship between ACEs and obesity and found an association between experiencing more ACEs and higher BMI [15–18]. Recent evidence from the Growing Up in Ireland study, which is very similar to GUS in design, found that ACEs up to the age of 9 were predictive of obesity at age 13 [19]. Although models controlled for caregiver BMI, as well as diet and exercise, they did not control for BMI in mid-childhood or maternal BMI in pregnancy. The ACEs instrument has been criticised as inherently imbalanced because it omits any consideration of positive childhood influences on later health and well-being. We, therefore, use ACEs measures in combination with GUS-collected proxies for established measures of “Protective Childhood Experiences (PCEs)” [20].

## Methods and materials

### Source of data and participants

The GUS cohort study is the largest Scottish cohort (longitudinal) study of children launched in the last two decades. This analysis used data from Birth Cohort 1 (*n* = 5217) born in 2004/5 with families first interviewed when the child was aged 10 months. These children and their families have been recurrently interviewed, examined and followed-up since infancy: a total of nine sequential face-to-face interviews have been conducted with each family to age 12, in 2016–17. Full details of the sampling and design of the GUS study can be found in the Data Documentation [21–23].

#### Ethical review

This project was approved in early 2020 by an expedited Usher Institute Research Ethics process, University of Edinburgh, since no contact with human subjects was involved, and all data were anonymised in the archived form they were received. As described in the Data Documentation [21–23], all subjects in the original GUS cohort study gave informed consent for their data to be collected, analysed for research purposes, including being anonymously linked to other datasets where required.

The age 12 GUS follow-up data include interviewer-measured height and weight at five separate ages, allowing the calculation of each subject’s weight-for-height, categorised according to standard percentile cut-offs from a historical UK population sample [24]: underweight; normal; overweight; obese.

#### Analyses and study power

We used multiple logistic regression to analyse age-12 weight-for-height in the “obese” category as our primary outcome, and previously collected data in the GUS cohort as our candidate predictor variables [11]. Our extensive experience analysing GUS data showed that the available number of children with complete weight-for-height data at age 12 (*N* = 2787—see below), despite study attrition, is still sufficiently large to identify “clinically significant” (i.e., OR > 1.2 or <0.8) predictors of persistent obesity at age 12, and provide reasonably precise estimates of their effects, for even relatively uncommon risk factors affecting only about one quarter of the cohort population.

As overall summary measures of best fit and most parsimonious model’s predictive validity, we analysed Nagelkerke’s R^2^, Harrell’s C-statistic, and the area under each final model’s Receiver Operator Characteristic Curve (AUROC), as well as the positive and negative predictive values of the model at the optimal sensitivity/specificity cutoff (maximising Youden’s Index, the sum of sensitivity and specificity, which assumes equal relative harms arising from false positive and false negative screens). Our analysis plan was therefore to produce the following outputs:

- the most sensitive/specific predictive algorithms for the primary outcome (obesity at age 12), using all the literature-derived candidate predictor variables in the full GUS dataset which were collected by age 6. Priority in selection of variables for initial model inclusion was given to life-course predictor variables (i.e., measured from prenatal life onwards), for which data are frequently routinely collected, machine-readable, and linkable at the population level in HICs - for example in perinatal and Health Visitor databases.
- a sensitivity analysis of which subsets of predictor variables, in the best fit and most parsimonious full models based on all GUS candidate predictors, perform best in national settings where data sources for those predictors are not universally available/machine-readable/linkable to other data for the purposes of population screening: perinatal data; Health Visitor data; and other routine data collection (e.g., age 5–6 BMI measured in schools, as in Scotland).

### Outcome

The main outcome is obesity at age 12, defined according to the Information Services Division Scotland, which uses UK growth reference standards to produce BMI centiles with standard cutoffs, following Cole’s method [25], and for population health monitoring purposes, defines child obesity as a BMI greater than or equal to 95th centile [26] of the historical population’s “normal” distribution.

### Predictors

Potential predictors were chosen based on previous research (see above), availability in the GUS cohort, and their feasibility to be collected routinely in HICs. These predictors were: (1) mother’s age at child’s birth (<20, 20–29, 30–39 ≥ 40 years age groups); (2) mother’s ethnicity (white vs other - Scottish population-based samples contain such small percentages of any given “non-White” ethno-racial group that they are generally combined to increase statistical power; (3) child’s birth order, (4) maternal smoking in pregnancy (yes vs no), (5) mother’s BMI as a continuous variable, measured in sweep 6 (when children were 5–6 years); (6) GDM or diabetes in mother’s pregnancy; (7) maternal education (initially classified according to the Scottish Credit and Qualifications Framework and categorised into: (a) higher or above, (b) standard grade/other, and (c) no qualifications); (8) location (referring to baseline geographical area dichotomised into: urban vs rural); (9) equivalized household income quintile at recruitment; (10) SIMD quintile of family residence address at recruitment; (11) household indoors smoking in the family home, assessed at sweeps 1 and 5–9; (12) whether the child was delivered by caesarean; (13) gestational age at birth (<3 weeks early vs ≥3 weeks early); (14) birthweight (<2500 g vs ≥2500 g); (15) breastfeeding (never, <6 months, and ≥6 months); (16) age at introduction to solid foods (dichotomised according to Scottish guidance at the time [27, 28] into: <4 months vs ≥4 months); (17) child’s sex; (18) child’s ethnicity (“white” vs “other” – typical Scottish population samples do not include sufficient numbers of non-white subjects to allow analysis by sub-ethnicity sub-categories, and the publicly available dataset therefore combines them); (19) child’s BMI, measured in sweep 6 at age 5–6, as continuous variable; (20) ACEs count—we were able to find proxy variables for seven out of ten ACEs: physical abuse, emotional neglect, domestic violence, substance misuse, mental illness, parent in prison, and separation; (21) PCEs count -proxy variables were found for five out of seven PCEs: “I am able to talk to my family about my feelings;” “my family stands by me during difficult times;” “I feel a sense of belonging in school;” “I feel supported by friends;” and, “at least two non-parent adults take genuine interest in me.” A fuller account of variable construction is available in Supplementary Material Part A.

### Sample size and missing data

**Fig. 1:**
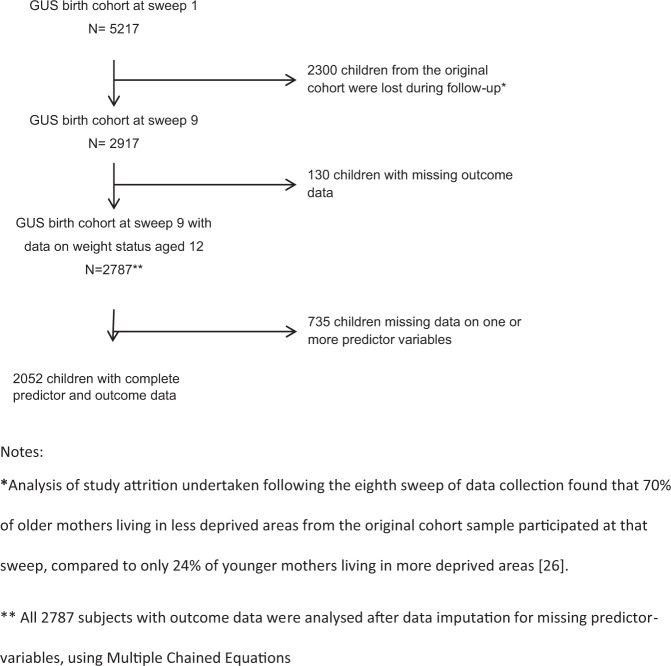
Flow chart – missingness of data by GUS study stage. Flow chart displaying missingness of data from initial sample at Sweep 1 to analytical sample at Sweep 9.

The analysis included 2787 children with complete outcome (height, weight, and age) data, out of 2917 subjects successfully followed up to data collection sweep 9 in the GUS cohort. Amongst these 2787 subjects, 26.2% (*n* = 735) had at least one potential predictor variable missing. Figure 1 depicts the process followed to select the final sample for analysis. An analysis of jointly missing data [see Supplementary Material Part B] indicated that missingness may well have resulted in bias. We, therefore, used Multiple Chained Equations [29], without any auxiliary variables, to impute missing data – creating 30 imputed datasets in total - for all 2787 GUS subjects with outcome data, the results of which are presented below.

### Statistical analysis

The GUS datasets have survey weights available, which are useful for estimating population averages corrected for sampling and – to a degree – attrition biases. However, it has been suggested that the use of survey weights in regression analyses may have limitations, such as increasing standard errors or providing unreliable coefficient estimates [30, 31]. In addition, the GUS datasets available from UKDS for longitudinal analyses supply weights based only on subjects who provided data on every sweep, whereas our analysis is based on all subjects with outcome data at age 12, so using those weights would have substantially reduced our sample size. Finally, key characteristics known to predict non-response/attrition in the GUS cohort – maternal age and education, family income and Index of Multiple Deprivation – were included in all our multivariate models, after data imputation. Therefore, weighted analyses were not performed.

First, bivariate analyses were conducted to assess the association between obesity at age 12 and each potential predictor. 16 of the 21 predictors in the initial “full model,” with a *p* < 0.1 were selected for further analysis in a multivariable model: (1) maternal age, (2) maternal BMI, (3) maternal education, (4) smoking in pregnancy, (5) GDM/diabetes in pregnancy, (6) location, (7) income quintile, (8) level of deprivation, (9) indoors smoking, (10) birth by Caesarean section, (11) breastfeeding (never, less than or more than six months duration), (12) introduction to solid food before 4 months of age, (13) child’s sex, (14) child’s BMI at age 5–6, (15) ACEs count, and (16) PCEs count.

Secondly, bivariate polychoric correlation analyses were conducted to determine whether some potential predictors were strongly enough associated to cause collinearity problems in the multivariate analyses. We were particularly interested in the correlation between household income and SIMD; no strong correlation between any pairs of covariates was found [see Supplementary Material Part C]. Then, predictor-variable reduction to obtain final models was performed by stepwise selection (backwards and forwards), with a cut-off *p*-value of *p* = 0.06 [32], also retaining any variables which caused more than a 10% change in other variables’ beta-coefficients when removed (this consideration, per se, added no variables to the final model). The “Optimal Data” model with the best fit consisted of six predictors. To avoid the use of predictor variables which are not routinely collected and/or machine readable in some settings, including Scotland, we excluded ACEs and PCEs from the initial multivariable ‘full model’ to create a second, final Scottish Data model. It also consisted of six predictors of which two differed from the six predictors in the Optimal Data model: Scottish Index of Multiple Deprivation quintile replaced equivalized income and “introduction of solids before age 4 months” replaced ACEs in the Scottish Data model. An internal validation was conducted for both final models, “Optimal Data Availability” and “Scottish Data”, by means of bootstrapping, with application of the resultant shrinkage factor to revised odds ratio estimates for all predictors [33].

Finally, the discriminatory performance of each internally validated model was assessed. This is reported at the bottom of Table 2 as Nagelkerke R^2^ and Harrell’s C-statistic for the Optimum Data and Scottish Data models, respectively. Sensitivity-Specificity and AUROC curves were plotted to show the full range of sensitivity and specificity. An optimal cut-off point was selected by Youden’s Index, which maximises the sum of Sensitivity and Specificity. Analyses were performed using R and Stata version 16. The key R packages used were: “mice”, “psfmi” and “ROCit” [34–36].

## Results

### Participants

For the 2787 children included in the imputed datasets, the maternal and child characteristics considered as potential predictors of obesity at age 12 are shown in Table 1. Just over half of the children were male, and the majority resided in urban areas. According to their BMI, 18.3% (393) of the children were in the obese category at age 12.

**Table 1:** Observed data.

*N* = 2787		n/mean	%/SD
Maternal age (years)	<20	88	3.2
	20–29	953	34.3
	30–39	1636	58.8
	≥40	104	3.7
	Missing	6	
Maternal BMI (Kg/m^2^)		26.9	5.6
	Missing	457	
Maternal education	Higher and above	2248	80.8
	Standard grade/other	378	13.6
	No qualifications	156	5.6
	Missing	5	
Smoked in pregnancy, yes		485	17.7
	Missing	46	
GDM/diabetes in pregnancy, yes		26	0.9
	Missing	0	
Location, urban		1842	66.1
	Missing	0	
Income quintile SW1	Top quintile	620	24.4
	4th quintile	636	25.0
	3rd quintile	505	19.9
	2nd quintile	472	18.6
	Bottom quintile	311	12.2
	Missing	243	
SIMD quintile SW1	Least deprived 1	655	23.5
	2	627	22.5
	3	599	21.5
	4	441	15.8
	Most deprived 5	465	16.7
	Missing	0	
Indoors smoking, yes		807	29.0
	Missing	0	
Child was born by caesarean	737	26.4	
	Missing	0	
Breastfeeding	≥6 months	840	30.2
	<6 months	1083	38.9
	Never	863	30.2
	Missing	1	
Introduction to solid foods, ≥4 months		2424	88.0
	Missing	32	
Child’s sex, male		1404	50.4%
	Missing	0	
BMI age 5–6	kg/m^2^	16.2	1.8
	Missing	172	
Obesity age 11–12		516	18.5
ACE – Physical abuse		491	18.4
	Missing	123	
ACE – Emotional neglect		560	21.3
	Missing	159	
ACE – Domestic violence		59	2.2
	Missing	80	
ACE – Mental illness		1024	36.7
	Missing	0	
ACE – Parent in prison		20	0.7
	Missing	0	
ACE – Parental separation	817	29.3	
	Missing	0	
ACEs count	0	830	29.8
	1	963	34.6
	2	591	21.2
	3	286	10.3
	4+	117	4.2
	Missing	0	
PCE – Share feelings with family		2010	72.5
	Missing	14	
PCE – Family support in difficult times		2624	94.6
	Missing	14	
PCE – Feel they belong in their school		1884	71.8
	Missing	162	
PCE – Friends support them		1695	61.4
	Missing	25	
PCE – Two non-parent adults		293	10.6
	Missing	27	
PCEs count	0–2	763	27.5
	3	898	32.7
	4–5	1.113	40.1
	Missing	13	

### Model development and specification

After conducting stepwise regression, the predictors included in the final models were very similar. Both models included maternal BMI, indoors smoking, caesarean delivery, child’s sex and child’s BMI at age 5–6. The final “Scottish Data” model, without ACEs as a predictor (included in the Optimum Data model), included SIMD instead of equivalized income, and age at introduction to solid foods, which were not in the final Optimum Data model.

Table 2 presents the regression coefficients and odds ratios (OR) for these two models. Internal validation by bootstrapping showed a shrinkage of 0.974 and 0.981 for the Optimum Data model and the Scottish Data model, respectively. On the basis of the respective shrinkage factors, all regression coefficients were recalibrated, as shown in Table 2.

**Table 2:** Final prediction models for obesity at age 12.

*N* = 2787		Optimum data model					Scottish data model				
		Before internal validation				After internal validation	Before internal validation				After internal validation
		B	OR	95% CI		B	B	OR	95% CI		B
Intercept		−17.182				−16.742	−16.622				−16.312
Maternal BMI (Kg/m^2^)^1^		0.070	1.07	1.05	1.10	0.068	0.070	1.07	1.05	1.10	0.068
Indoors smoking^2^	No		1.00					1.00			
	Yes	0.313	1.37	1.05	1.78	0.305	0.463	1.59	1.23	2.05	0.454
Equivalized income^1^	Top quintile	−0.224	0.80	0.51	1.25	−0.218					
	4th quintile	−0.255	0.77	0.51	1.19	−0.249					
	3rd quintile	−0.007	0.99	0.66	1.50	−0.007					
	2nd quintile	0.237	1.27	0.85	1.89	0.231					
	Bottom quintile		1.00								
SIMD^1^	Q1 (least deprived)							1.00			
	Q2						0.419	1.52	1.05	2.20	0.411
	Q3						0.304	1.35	0.93	1.98	0.298
	Q4						0.386	1.47	0.98	2.20	0.378
	Q5 (most deprived)						0.571	1.77	1.20	2.62	0.560
Introduction to solid foods^2^	<4 months							1.00			
	≥4 months						−0.355	0.70	0.51	0.96	-0.349
Child’s sex^1^	Female		1.00					1.00			
	Male	0.507	1.66	1.31	2.11	0.494	0.509	1.66	1.31	2.10	0.499
BMI age 5–6 (Kg/m^2^)^2, 3^		0.781	2.18	2.00	2.38	0.761	0.765	2.15	1.97	2.34	0.751
ACEs count^4^	0		1.00								
	1	0.321	1.38	1.00	1.90	0.313					
	2	0.709	2.03	1.43	2.89	0.691					
	3	0.857	2.36	1.55	3.58	0.835					
	4+	0.853	2.35	1.31	4.19	0.831					
*Nagelkerke R*^*2*^		0.384				0.374	0.373				0.364
*C statistic*		0.855				0.851	0.848				0.845

^1,2,3,4^The data sources for these predictors are, in typical high-income countries: 1: Pre/Perinatal Dataset; 2: Health Visitor Dataset; 3: Primary School Dataset; 4: New primary data collection.

**Fig. 2:**
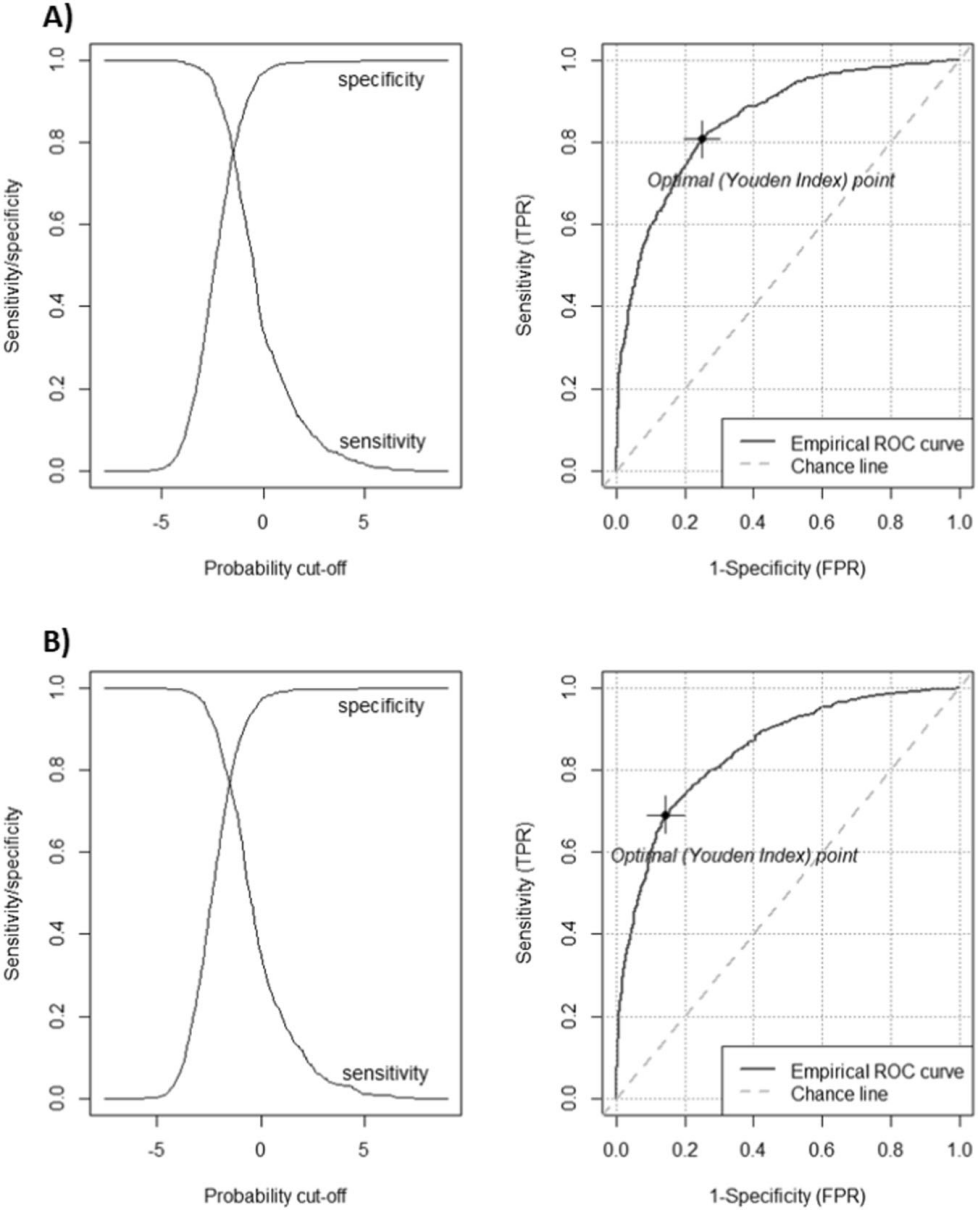
Sensitivity/specificity plots and receiver operator curves. **A** “Optimum Data Availability” and **B** “Scottish Data” models.

Sensitivity-Specificity and AUROC curves were plotted (Fig. 2) to test the two internally validated models’ predictive validity over the full range of sensitivity and specificity cut-offs. Then, Youden’s index was calculated; the cut-off thereby selected, by maximising the sum of Sensitivity and Specificity, had a Youden’s index of 0.217, AUROC = 0.855 (95% CI 0.852–0.859) for the Optimum Data model, with a Youden’s index was 0.226, AUROC = 0.849 (95% CI 0.846–0.852) for the Scottish Data model, as defined by the predicted probability of the outcome. Table 3 presents the two-by-two screening-test validity tables for this cut-off point, for both final models.

**Table 3:** Two-by-two table of prediction models’ validity at selected cut-off.

		“Optimum Data Availability” Cut-off: 0.217			“Scottish Data” Cut-off: 0.226			
	*N* = 2118	True (observed) outcome			*N* = 2279	True (observed) outcome		
		Obese	Non-obese	TOTALS		Obese	Non-obese	TOTALS
Predicted outcome	Obese	297	387	784	Obese	314	388	702
	Non-obese	92	1342	1434	Non-obese	98	1479	1577
	TOTALS	389 (Obese)	1729 (Non-Obese)		TOTALS	412 (Obese)	1867 (Non-Obese)	
	Sensitivity:	297/389 =	76.3%		Sensitivity:	314/412=	76.2%	
	Specificity:	1342/1729 =	77.6%		Specificity:	1479/1867=	79.2%	
	PPV	297/784=	37.8%		PPV:	314/702=	44.7%	
	NPV	1342/1434=	93.6%		NPV:	1479/1577=	93.8%	
	Referral Burden:	784/2118 =	37.0%		Referral Burden:	702/2279 =	30.8%	

Of the 387 “false positives” from the Optimal data model 160 (41.4%) were overweight, so that those unlikely to benefit at all would be 227 or 10.7% of all those screened; of the 388 “false positives” from Scottish data model 161 (41.5%) were overweight, so that those unlikely to benefit at all would be 227 or 10.0% of all those screened.

*PPV* positive predictive value, *NPV* negative predictive value.

The Optimum Data model’s performance, as shown in Table 3, misclassified 18.3% (387/2118) of the children as “false positives” i.e., predicted to be obese at age 12 when the observed outcome was non-obesity. However, further inspection revealed that 41.4% (160) of these 387 “misclassified” children were in fact overweight at age 12, and thus could potentially benefit from referral to a specialist care at age 5–6. The Scottish Data model misclassified 17.0% (388/2279) of the screened children as false positives; however, additional inspection showed that 41.5% (161) of these 388 children were overweight at age 12. Effectively, this means that the children identified as at risk of future obesity or overweight by the screening, who are “indisputable false positives” (in that they will not be either obese or overweight at age 12, and therefore unlikely to benefit at all from referral at age 5–6) would amount to only 10.7% (Optimal Data model) to 10.0% (Scottish Data model) of all those screened.

## Discussion

The results above indicate that a universal obesity-risk screening programme at age 5–6, as part of a national obesity surveillance system, would be able to detect over three-quarters (for both of the two models) of the 18% of Scottish children destined to be obese at age 12, as well as another 7.6% (Optimum Data model) or 7.1% (Scottish Data model) of those screened who will be overweight at age 12 – all of whom could potentially benefit from early referral at age 5–6, before overweight or obesity is well established. However, the “cost” – in terms of referral burden – of this screening would be the identification of about one-third (37% in the Optimum Data model, 31% in the Scottish Data model) of all 5-to-6-year-olds as “at risk.”

In the present Scottish context, such a referral burden would likely not be supportable by the existing referral networks in primary care. Moreover, even if a screening test has shown reasonable sensitivity and specificity, as well as “total test positives” (referral burden) at the prevailing risk prevalence, does not mean screening should proceed. The full set of criteria for making such a decision are well documented in the pioneering publications by Wilson and Jungner, and Raffle and Muir Gray [37, 38]. In particular, evidence would be needed on any potentially harmful “labelling effects” of such screening on children screened positive, and on the long-term effectiveness of treatment on referral.

Predictors of pre-adolescent obesity in this study were mostly consistent with the previous literature: maternal BMI, particularly if the mother was obese during pregnancy, and having higher levels of ACEs were the strongest predictors of pre-adolescent obesity. “Obesity engenders obesity” was the conclusion of a recent review exploring the impact of maternal obesity in pregnancy on child metabolic outcomes, with heavier women giving birth to heavier babies, who in turn are more likely to become overweight or obese [39], whilst high levels of ACEs has been consistently associated with subsequent obesity [15–18]. The child’s BMI at age 5–6 was additionally associated with later obesity, in line with other findings on obesity throughout childhood [40], as well as having been born by Caesarean section, and being male, which have also been found to be predictive of obesity in other studies [41]. A household member smoking inside the home was also associated with higher risk for child obesity, and this association appeared stronger than that for smoking in pregnancy, which was not statistically significant in our adjusted model: previous evidence, however, suggests stronger effects of maternal smoking in pregnancy even after controlling for other passive smoking effects, such as father’s smoking [42]. These studies do not appear to have controlled for whether the mother continued to smoke in the household after birth. Overall, these predictive factors point to a range of family environmental and potentially genetic factors which are playing a role in levels of childhood obesity being seen in many HICs. This familial background is important to consider when planning interventions for childhood obesity.

Our purpose, however, in assessing the predictive validity of routinely collected early-to-mid-childhood variables for obesity at age 12, was purely to quantify these variables’ multivariable predictive validity. The Scottish Data model, not including ACEs as a predictor, is slightly more predictive, in terms of its superior specificity (79.2%, versus 77.6% for the Optimum Data model) and lower referral burden (30.8% versus 37%). We hope that future work will now be able to pilot actual screening programmes, with full and robust evaluation of all the consequences, including potentially harmful “labelling” effects in both true and false positives, and any false reassurance effects to future obesity cases missed by the screen, as well as the all-important frequency and consequences of referral/treatment failure. Then a full ethical, logistical and health economic analysis is warranted to determine the full pros and cons of such a screening programme.

### Study strengths

This study uses data from a large birth cohort, which was sampled to be representative of the population. These data are far richer than those from routinely collected data sources, or growth-based cohorts, and include repeated height and weight data which were directly measured.

### Study limitations

As is typical in cohort studies, GUS suffers from differential attrition, which means that it is more likely to lose families from the most deprived backgrounds, as already noted. While some researchers might attempt to correct for resulting attrition biases, we are of the view that there are no entirely satisfactory methods for doing so within the analytical approaches used here. As our models included key subject characteristics known to predict non-response/attrition in the GUS cohort (e.g., maternal age and education, family income, and SIMD) and we used data imputation based on Multiple Chained Equations, the impact of attrition bias on our results should be limited. Furthermore, we note that in both our final models (Table 2) maternal education did not appear as an independent predictor, and equivalized income was not statistically significant in the Optimum Data Model (although SIMD showed statistically significant effects for Q2 and Q5 in the Scottish Data Model.)

In addition, many of the predictive factors are self-reported, and thus may be affected by social desirability bias. Breastfeeding and birth data were collected at Sweep 1 when children were 10 months old and may therefore be affected by recall bias.

An important consideration for those wanting to use our results in an actual screening programme is the extent to which our two final predictive models (Optimum Data and Scottish Data) involve major differences in routine data collection and its associated costs, as well as their intrusiveness/respondent burden. On reflection, we suspect that even our limited proxy-set of ACE indicators is not feasible to collect universally in most settings, even in HICs, because some of the questions are so sensitive, potentially interfering with the often-delicate relationship between Health Visitors/Community Nurses and high-risk families. This obviously implies that the Scottish Data model - relying as it does on the question “At what age were solid foods begun?” – rather than ACE indicators, is much more feasible data collection strategy for the prediction of future obesity risk. As well, the slightly higher specificity of the Scottish Data model (79.2% versus 77.6% for the Optimum Data model), leads to a more manageable referral burden (31% versus 37% of all children screened, respectively), at virtually identical sensitivities (76.3% versus 76.2%, respectively).

## Conclusions

As the first step in that necessarily long and deliberative process, we believe this study has demonstrated that an acceptable level of predictive validity for obesity at age 12 can be achieved very cost-effectively, using only a half-dozen predictor variables which are routinely collected before age 6 in many countries. These analyses will inform the design of future National Obesity Surveillance Systems for any similar setting—i.e., countries, mostly high-income, which have for some decades had a significant public health problem of child obesity. Such systems should ideally not only measure (and monitor over time) the magnitude of these health outcomes and their trends. These systems should also facilitate early identification of the highest-risk children and their families, before persistent and/or severe excess weight problems develop, as priority targets for cost-effective, early, treatment and prevention interventions delivered to children at high risk, well before full-blown obesity is established. However, the quantification of such risk-prediction algorithms’ predictive validity is but one of many evidentiary elements required to justify the implementation of such programmes, given the considerable uncertainties around referral-system capacity, long-term treatment efficacy, effectiveness, and efficiency, as well as the potential side-effects of screening (especially labelling effects in false positives). Perhaps most importantly, the high prevalence of obesity at age 12 in this cohort (18.3%) meant that using even the more specific Scottish Data model would lead to nearly a third of children being referred for specialist treatment – likely an unsupportable referral burden in even the wealthiest countries.

## Supplementary information

Validity of Scottish Predictors of Child Obesity (age 12) for Risk Screening in Mid-Childhood (age 5–6): A Prospective Cohort Study SUPPLEMENTARY DATA

## Data Availability

All data analysed in this study are available on request to the UK Data Archive. Due to the complexity of the analyses in this study, involving several sorts of software, all computer codes utilised are available on request, by email to the corresponding author.

## References

[1] Blake-Lamb TL, Locks LM, Perkins ME, Woo Baidal J, Cheng ER, Taveras EM (2016). Interventions for childhood obesity in the first 1,000 days: a systematic review. Am J Prev Med.

[2] Redsell SA, Edmonds B, Swift JA, Siriwardena AN, Weng S, Nathan D (2016). Systematic review of randomised controlled trials of interventions that aim to reduce the risk, either directly or indirectly, of overweight and obesity in infancy and early childhood. Matern Child Nutr.

[3] Pandita A, Sharma D, Pandita D, Pawar S, Tariq M, Kaul A (2016). Childhood obesity: prevention is better than cure. Diabetes Metab Syndr Obes.

[4] Canfell OJ, Littlewood R, Wright ORL, Walker JL. Clinical relevance and validity of tools to predict infant, childhood and adulthood obesity: a systematic review. Public Health Nutr, 2018. Published online: 12 July 2018. 10.1017/S1368980018001684. 10.1017/S1368980018001684 PMC10260762 29996950

[5] Ziauddeen N, Roderick PJ, Macklon NS, Alwan NA (2018). Predicting childhood overweight and obesity using maternal and early life risk factors: a systematic review. Obes Rev.

[6] Butler É, Derraik M, Taylor JG, Cutfield RW (2018). W.S., Prediction models for early childhood obesity: applicability and existing issues. Horm Res Paediatr.

[7] Singh AS, Mulder C, Twisk JW, Van Mechelen W, Chinapaw MJ (2008). Tracking of childhood overweight into adulthood: a systematic review of the literature. Obes Rev.

[8] Baidal JAW, Locks LM, Cheng ER, Blake-Lamb TL, Perkins ME, Taveras EM (2016). Risk factors for childhood obesity in the first 1,000 days: a systematic review. Am J Prev Med.

[9] Welten M, Wijga AH, Hamoen M, Gehring U, Koppelman GH, Twisk JWR (2020). Dynamic prediction model to identify young children at high risk of future overweight: Development and internal validation in a cohort study. Pediatr Obes.

[10] Doi L, Williams AJ, Frank J (2016). How has child growth around adiposity rebound altered in Scotland since 1990 and what are the risk factors for weight gain using the Growing Up in Scotland birth cohort 1?. BMC Public Health.

[11] Bradshaw P. Growing Up in Scotland: Overweigh and Obesity at Age 10. 2018, Scottish Government: Edinburgh.

[12] Felitti VJ, Anda RF, Nordenberg D, Williamson DF, Spitz AM, Edwards V (1998). Relationship of childhood abuse and household dysfunction to many of the leading causes of death in adults. The Adverse Childhood Experiences (ACE) Study. Am J Prev Med.

[13] Marryat L, Frank J (2019). Factors associated with adverse childhood experiences in Scottish children: a prospective cohort study. BMJ Paediatrics Open.

[14] Blair A, Marryat L, Frank J (2019). How community resources mitigate the association between household poverty and the incidence of adverse childhood experiences. Int J Public Health.

[15] McKelvey LM, Saccente JE, Swindle TM (2019). Adverse childhood experiences in infancy and toddlerhood predict obesity and health outcomes in middle childhood. Child Obes.

[16] Fuemmeler BF, Dedert E, McClernon FJ, Beckham JC (2009). Adverse childhood events are associated with obesity and disordered eating: results from a U.S. population-based survey of young adults. J Trauma Stress.

[17] Isohookana R, Marttunen M, Hakko H, Riipinen P, Riala K (2016). The impact of adverse childhood experiences on obesity and unhealthy weight control behaviors among adolescents. Compr Psychiatry.

[18] Rehkopf DH, Headen I, Hubbard A, Deardorff J, Kesavan Y, Cohen AK (2016). Adverse childhood experiences and later life adult obesity and smoking in the United States. Ann Epidemiol.

[19] Gardner R, Feely A, Layte R, Williams J, McGavock J (2019). Adverse childhood experiences are associated with an increased risk of obesity in early adolescence: a population-based prospective cohort study. Pediatr Res.

[20] Baumrind D. The Development of Instrumental Competence through Socialization, in Minnesota Symposia on Child Psychology: 7, A Pick, Editor. 1973, University of Minnesota Press:Minneapolis. p. 3–46.

[21] Vosnaki K, Bradshaw P, Scholes A, Life at age 12: Initial findings from the Growing Up in Scotland study. 2019, Scottish Government: Edinburgh.

[22] Public Health Information for Scotland. Growing Up in Scotland. 2019; Available from: https://www.scotpho.org.uk/publications/overview-of-key-data-sources/surveys-longitudinal/growing-up-in-scotland.

[23] Bradshaw P, Tipping S, Marryat L, Corbett J, Growing Up In Scotland Sweep 1 - 2005: User Guide. 2005, Scottish Centre for Survey Research: Edinburgh.

[24] Dinsdale H, Ridler C, Ells L. A Simple Guide To Classifying Body Mass Index In Children. 2011, National Obesity Observatory: Oxford.

[25] Cole TJ, Green PJ (1992). Smoothing reference centile curves: the LMS method and penalized likelihood. Stat Med.

[26] ScotCen Social Research, Growing Up in Scotland Sweep 9: 2017–18 User Guide. 2018, Scottish Centre for Survey Research: Edinburgh.

[27] Department of Health. Weaning and the Weaning Diet Report of the Working Group on the Weaning Diet of the Committee on Medical Aspects of Food Policy. 1994, Department of Health: London. 7701106

[28] Alder EM, Williams FL, Anderson AS, Forsyth S, Florey CD, Van der Velde P (2004). What influences the timing of the introduction of solid food to infants?. Br. J. Nutr.

[29] Heymans MW, Eekhout I, Applied missing data analysis with SPSS and (R)Studio. 2019, R Bookdown: Amsterdam, Available online: https://bookdown.org/mwheymans/bookmi/.

[30] Heeringa SG, West BT, Berglund PA. Applied Survey Data Analysis. 2017, CRC Press LLC: Milton, UK.

[31] Gelman A (2007). Struggles with survey weighting and regression modeling. Stat Sci.

[32] Steyerberg E. Clinical Prediction Models: A Practical Approach to Development, Validation, and Updating. 2nd. ed. Statistics for Biology and Health. 2019, p. 297-308, Springer International Publishing: Cham, Switzerland.

[33] Schomaker M, Heumann C (2018). Bootstrap inference when using multiple imputation. Stat Med.

[34] van Buuren S, Groothuis-Oudshoorn K. Mice: Multivariate imputation by chained equations in R. J Stat Software. 2011. 45p. 1–67. Available from: https://www.jstatsoft.org/v45/i03/.

[35] Heymans M, psfmi: Prediction model pooling, selection and performance evaluation across multiply imputed datasets. R package version 1.0.0. 2021. Available from: https://CRAN.R-project.org/package=psfmi Available online: https://bookdown.org/mwheymans/bookmi/

[36] Khan RA, Brandenburger, T. ROCit: Performance Assessment of Binary Classifier with Visualization. R package version 2.1.1. 2020. Available from: https://CRAN.R-project.org/package=ROCit

[37] Wilson JMG, Jungner G, Principles and Practice of Screening for Disease. 1968, World Health Organization: Geneva.

[38] Raffle AE, JM Gray, Screening: Evidence and Practice. 2019, Oxford University Press: New York, USA.

[39] Valsamakis G, Kyriazi EL, Mouslech Z, Siristatidis C, Mastorakos G (2015). Effect of maternal obesity on pregnancy outcomes and long-term metabolic consequences. Hormones (Athens).

[40] Wright CM, Marryat L, McColl J, Harjunmaa U, Cole TJ (2018). Pathways into and out of overweight and obesity from infancy to mid-childhood. Pediatr Obes.

[41] Bammann K, Peplies J, De Henauw S, Hunsberger M, Molnar D, Moreno LA (2014). Early life course risk factors for childhood obesity: the IDEFICS case-control study. PLoS One.

[42] Riedel C, Schönberger K, Yang S, Koshy G, Chen YC, Gopinath B (2014). Parental smoking and childhood obesity: higher effect estimates for maternal smoking in pregnancy compared with paternal smoking-a meta-analysis. Int J Epidemiol.

