# Supplementary material for: Validity of Scottish predictors of child obesity (age 12) for risk screening in mid-childhood: a secondary analysis of prospective cohort study data—with sensitivity analyses for settings without various routinely collected predictor variables
Source: Int J Obes (Lond). 2022 Jun 3;46(9):1624–32. doi: 10.1038/s41366-022-01157-5 (PMC9395267; doi:10.1038/s41366-022-01157-5)
Supplement: Supplementary file 1 — Validity of Scottish Predictors of Child Obesity (age 12) for Risk Screening in Mid-Childhood (age 5–6): A Prospective Cohort Study SUPPLEMENTARY DATA [file 41366_2022_1157_MOESM1_ESM.docx]

Validity of Scottish Predictors of Child Obesity (age 12) for Risk Screening in Mid-Childhood (age 5-6): A Prospective Cohort Study

**SUPPLEMENTARY DATA**

# Candidate predictors

Table S1 below shows the all the variables included in this study and how they were built. Variables’ operationalisation includes the name of the variable as specified in the GUS cohort and the sweep in which the variable was taken.

### Table S1. Variable operationalisation.

|  | | | | | Sweep | | Variable | | | | Label | |
| --- | --- | --- | --- | --- | --- | --- | --- | --- | --- | --- | --- | --- |
| **Maternal factors** | | | | | | | | | | | | |
| Demographics | Age | | | | 1 | | DaHGmag5 | | | | Age of mother at birth of sample child | |
|  | Ethnicity | | | | 1 | | DaEthGpM  DaEthGpP | | | | Ethnicity of Respondent (if mother) OR  Ethnicity of Partner (if respondent=father) | |
|  | Location | | | | 1 | | ALaURin2 | | | | SE urban-rural classification | |
| Parity | | | | | 1 | | DaHGbord | | | | Study child’s birth order | |
| Smoking | | | | | 1 | | MaHcig01 | | | | Smoke during pregnancy | |
| BMI | | | | | 6 | | DfMbmig5 | | | | Valid BMI | |
| GDM | | | | | 1 | | MaPGil01 | | | | During your pregnancy, did you have any illnesses or other problems that required medical attention or treatment? | |
|  |  |  |  |  | 1 | | MaPGil07 | | | | Diabetes during pregnancy? | |
|  |  |  |  |  | 1 | | MaPGil17 | | | | GDM/ raised blood sugar, abnormal OGTT results | |
| SES/  Education | Income | | | | 1 | | DaEqv5 | | | | Equivalised income ‐ quintiles | |
|  | SIMD | | | | 1 | | ALaSNimd | | | | SIMD quintiles | |
|  | Education | | | | 1 | | DaMedu01/ DaMedu02/ DaMedu03/ DaMedu04/ DaYedu04 | | | | Highest Education level (SCQF) | |
| **Birth factors** | | | | | | | | | | | | |
| Caesarean | | | | 1 | | | | MaBdel01 | | Type of delivery | | |
|  |  |  |  |  |  |  |  | MaBdel02 | | Type of delivery – if asked to partner | | |
| Gestational Age (GA) at birth | | | | 1 | | | | MaBtim01 | | Born early/late/on time | | |
|  |  |  |  |  |  |  |  | MaBtim02 | | How many days early | | |
|  |  |  |  |  |  |  |  | MaBtim03 | | How many weeks early | | |
| Birthweight & Weight for GA | | | | 1 | | | | DaWgGr | | Birthweight in grams | | |
|  |  |  |  |  |  |  |  | DaLwBWt | | Low birthweight | | |
| **Infant feeding factors** | | | | | | | | | | | | |
| Breastfeeding | | | | 1 | | MaBFDe01 | | | | | | Was child ever breastfed? |
|  |  |  |  |  |  | MaBFDs01 | | | | | | Still breastfeeding |
|  |  |  |  |  |  | MaBFDm01 | | | | | | Age (months) when stopped breastfeeding |
| Introduction of solid food | | | | 1 | | MaFsol02 | | | | | | Childs age in weeks when started solid food |
| **Child’s Factors** | | | | | | | | | | | | |
| Age | | | 9 | | | | DiHGag1 | | Age at interview in years | | | |
|  |  |  |  |  |  |  | DiHGagC | | Age at interview in months | | | |
| BMI | | | 6 | | | | DfBMI | | BMI | | | |
| Sex | | | 9 | | | | MiHGsx1 | | Study child’s sex | | | |
| Ethnicity | | | 1 | | | | DaEthGpC | | Ethnicity of the child | | | |
| Parental weight perception | | | 9 | | | | MiHwtc02 | | Which of these best describes study child? | | | |
| **ACEs** | | | | | | | | | | | | |
| Sub-classification | | Components | | | | | | | | | | |
| Physical abuse | | Sweep 7  CgPa3 My parents smack me when I have done something wrong = often/always  AND/OR  MgAPQ13 You slap your child when he has done something wrong = often/always | | | | | | | | | | |
| Emotional neglect | | Sweep 7  CgPa2 My parents ask about my day in school (never)  CgPa4 My parents play games or do other fun things with me (never)  CgPa6 My parents help me with my homework (never)  CgPa7 My parents tell me when I’m doing a good job with something (never)  CgPa13 My parents check to make sure I’m doing ok (never)  AND/OR  Sweep 9  MiPall I listen to what the study child has to say (never)  MiPAlb If I know something is bothering the study child, I ask him/her about it (never)  MiPAla I pay attention to the study child, even when I am busy (never)  CiMum1/ CiMumAl1/CiDad1/ CiDadAl1 My Mum/mum figure/dad/dad figure listens to what I have to say (never)  CiMum6/ CiMumAl6/CiDad6/ CiDadAl6 If my Mum/mum figure/dad/dad figure knows something is bothering me, she asks me about it (never)  CiMum8/ CiMumAl8/ CiDad8/ CiDadAl8 My Mum/mum figure/dad/dad figure pays attention to me (never) | | | | | | | | | | |
| Domestic Violence | | IF  MfHdv061 Since the child was born, has any partner or ex-partner ever pushed you or held you down? (Yes & Four or more times)  MfHdv062 Since the child was born, has any partner or ex-partner ever kicked, bitten or hit you? (Yes & Four or more times)  MfHdv063 Since the child was born, has any partner or ex-partner ever choked or tried to strangle/smother you? (Yes & Four or more times)  MfHdv064 Since the child was born, has any partner or ex-partner ever used a weapon against you, for example an ashtray or a bottle? (Yes & Four or more times)  MfHdv065 Since the child was born, has any partner or ex-partner ever forced you or tried to force you to take part in any sexual activity when you did not want to? (Yes & Four or more times)  AND  MfHdv07 How many times since study child was born have any of these things happened to you? (3= four or five, 4= six or more, 5= too many to count)  OR  MfHdv034 Partner ever threatened responded with weapon (Yes) | | | | | | | | | | |
| Substance misuse | | Alcohol  *Sweep 1, 3 & 5:* In an average week, how many units do you drink? (14 or more)  *Sweep 9 :* AUDIT C score of:  (MiHalc05 How often do you have a drink that contains alcohol + MiHalc06 How many units of alcohol do you have on a typical day when you are drinking? + MiHalc10 How often do you have 6 or more units of alcohol on one occasion?)  Drugs  *Sweep 1:* MaHdrg11 /MaHdrg12 /MaHdrg13 /MaHdrg14 /MaHdrg15 /MaHdrg16 /MaHdrg17 /MaHdrg18 /MaHdrg19 /MaHdrg20 Have you ever taken… in the last 12 months? Cannabis/ Amphetamines/ Cocaine /Crack /Ecstasy /Heroin /Methadone /LSD /Another illegal drug  *Sweep 3 & 5:* McDRGw01/ McDRGw02/ McDRGw03/ McDRGw04/ McDRGw05/ McDRGw06/ McDRGw07/ McDRGw08/ McDRGw09/ McDRGw010 / MeDRGw01/ MeDRGw02/ MeDRGw03/ MeDRGw04/ MeDRGw05/ MeDRGw06/ MeDRGw07/ MeDRGw08/ MeDRGw09/ MeDRGw010 When was the last time you took any of the following? Cannabis/ Amphetamines/ Cocaine or coke/ Crack/ Ecstasy /Heroin /Methadone prescribed by a doctor/ Street methadone/Acid or LSD/ Another illegal drug In the last month/ In the last year  Both  *Sweep 6 to 8:* Since last sweep, can I check, has the child experienced any: Drug taking/alcoholism in the immediate family (Yes) | | | | | | | | | | |
| Mental illness | | *Sweep1, 3, 5, 8*  DaSf12mn/ DcSf12mn/ DcSf12mn/ DhSf12mn Mental MCS-12 Scale = <36  AND/OR  *Sweep2 & 4*  IF ZDbHdas02/ ZDdHdas02 >1 AND/OR ZDbPdas02/ ZDdPdas02 Standardised DASS depression z-score >1  AND/OR  *Sweep 6, 7, 9*  MfOve16/ MgOve16/ MiOve16 Since last sweep, can I check, has the child experienced any:  Mental disorder in the immediate family = 1 | | | | | | | | | | |
| Parent in prison | | *Sweep2*  IF MbHGawa01 respondent or partner have been away from child =1 AND MbHGawa03 What was the reason?=5  AND/OR  *Sweep 3 to 5*  IF McHGaww1/ MdHGaww1/ MeHGaww1 respondent/spouse/partner have been away from child=1 AND (McHGaww3/ MdHGaww3/ MeHGaww3 What was the reason? =5 OR McHGaww4/ MdHGaww4/ MeHGaww4 What was the reason? =5)  AND/OR  *Sweep 6, 7 & 9*  MfOve20/ MgOve20/ MhOve20/ MiOve20 Since last sweep – parent in prison?= 1 | | | | | | | | | | |
| Separation | | *Sweep 1 to 9*  DaHGnp01/ DbHGnp01/ DcHGnp01/ DdHGnp01/ DeHGnp01/ DfHGnp01/ DgHGnp01/ DhHGnp01/ DiHGnp01 Number of natural parents in the household = 1 | | | | | | | | | | |
| **PCEs** | | | | | | | | | | | | |
| (1) I am Able to talk to family about feelings | | *Sweep 8*  ChMum7 / ChMumAl7 / ChDad7/ ChDadAl7 I share my thoughts and feelings with my Mum/Dad/resident mother/father figure (4=always true)  *Sweep 9*  CiMum7 / CiMumAl7 / CiDad7/ CiDadAl7 I share my thoughts and feelings with my Mum/Dad/resident mother/father figure (4=always true) | | | | | | | | | | |
| (2) My family stands by me during difficult times | | *Sweep 8*  ChMum3/ ChMumAl3/ ChDad3/ ChDadAl3 I can count on my Mum/Dad/^ resident mother/father figure to help me when I have a problem (4=always true)  *Sweep 9*  CiMum3/ CiMumAl3/ CiDad3/ CiDadAl3 I can count on my Mum/Dad/^ resident mother/father figure to help me when I have a problem (4=always true) | | | | | | | | | | |
| (3) I feel a sense of belonging in school | | *Sweep 8*  ChFeel5 How do you feel about the school you go to? (1=very happy) | | | | | | | | | | |
| (4) I feel supported by friends | | *Sweep 9*  CiCrFrl My friends listen to what I have to say. (4=always)  CiCrFrc I can count on my friends to help me when I have a problem (4=always) | | | | | | | | | | |
| (5) I have at least 2 non-parent adults who take genuine interest in me | | *Sweep 9*  CiSup Most people feel worried from time to time. What do you do if you are worried about something? 4=Talk to another relative/ 7=Talk to a teacher/ 8=Talk to someone else.  Someone else included doctor (3), counsellor (10), coach or leader of a club (11), a neighbour (7), a friend of the family (47), a friend’s family member (13), someone else (64). | | | | | | | | | | |

# Missing data

Before analysing the relationship between the outcome variable and the candidate predictors, an analysis of the completeness of the data was conducted. In table S2 is shown the proportion of missing data for each variable which was not complete. The variable with most missing values was maternal BMI (16.4%) which was measured only in SW6. Income had 8.7%.

*Table S2. Missing data in potential predictors*

| No. | Variable | Count | % |
| --- | --- | --- | --- |
| 1 | Maternal age | 6 | 0.2 |
| 2 | Maternal BMI | 457 | 16.4 |
| 3 | Maternal educational level | 5 | 0.2 |
| 4 | Smoking in pregnancy | 46 | 1.7 |
| 5 | GDM/diabetes in pregnancy | - | - |
| 6 | Location | - | - |
| 7 | Household Income | 243 | 8.7 |
| 8 | SIMD quintile | - | - |
| 9 | Indoors smoking | - | - |
| 10 | Caesarean | - | - |
| 11 | Breastfeeding | 1 | <0.1 |
| 12 | Introduction of solid food | 32 | 1.1 |
| 13 | Child’s sex | - | - |
| 14 | Child’s BMI at age 5-6 | 172 | 6.2 |
| 15 | ACEs count | - | - |
| 16 | PCEs count | 13 | 0.5 |

Overall, 73.6% (2,052) of children with complete outcomes had complete data for all other variables of interest. An analysis of missing patterns (table S3) was conducted for children with missing data to determine if data were jointly missing. As previously mentioned, 2,052 cases had complete data, in 261 cases the only missing variable was maternal weight status, 181 cases had data available for all variables except household income. In table S3 is presented the patterns of missing data and the number of cases for each pattern.

*Table S3. Missing patterns tabulated*

| No. | Variable |
| --- | --- |
| 1 | Maternal age |
| 2 | Maternal BMI |
| 3 | Maternal educational level |
| 4 | Smoking in pregnancy |
| 7 | Household Income |
| 11 | Breastfeeding |
| 12 | Introduction of solid food |
| 14 | Child’s BMI at age 5-6 |
| 16 | PCEs count |

| Variable  No. Cases | 1 | 2 | 3 | 4 | 7 | 11 | 12 | 14 | 16 |
| --- | --- | --- | --- | --- | --- | --- | --- | --- | --- |
| 2052 |  |  |  |  |  |  |  |  |  |
| 261 |  |  |  |  |  |  |  |  |  |
| 181 |  |  |  |  |  |  |  |  |  |
| 126 |  |  |  |  |  |  |  |  |  |
| 33 |  |  |  |  |  |  |  |  |  |
| 25 |  |  |  |  |  |  |  |  |  |
| 25 |  |  |  |  |  |  |  |  |  |
| 21 |  |  |  |  |  |  |  |  |  |
| 18 |  |  |  |  |  |  |  |  |  |
| 9 |  |  |  |  |  |  |  |  |  |
| 6 |  |  |  |  |  |  |  |  |  |
| 5 |  |  |  |  |  |  |  |  |  |
| 3 |  |  |  |  |  |  |  |  |  |
| 2 |  |  |  |  |  |  |  |  |  |
| 2 |  |  |  |  |  |  |  |  |  |
| 2 |  |  |  |  |  |  |  |  |  |
| 2 |  |  |  |  |  |  |  |  |  |
| 2 |  |  |  |  |  |  |  |  |  |
| 1 |  |  |  |  |  |  |  |  |  |
| 1 |  |  |  |  |  |  |  |  |  |
| 1 |  |  |  |  |  |  |  |  |  |
| 1 |  |  |  |  |  |  |  |  |  |
| 1 |  |  |  |  |  |  |  |  |  |
| 1 |  |  |  |  |  |  |  |  |  |
| 1 |  |  |  |  |  |  |  |  |  |
| 1 |  |  |  |  |  |  |  |  |  |
| 1 |  |  |  |  |  |  |  |  |  |
| 1 |  |  |  |  |  |  |  |  |  |
| 1 |  |  |  |  |  |  |  |  |  |
| 1 |  |  |  |  |  |  |  |  |  |

# Correlation analysis

Table S4 presents the bivariate correlations for all predictors included in the regression models. Some variables were significantly associated; however, most of them showed a weak association.

*Table S4. Polychoric correlation matrix for bivariate correlations between selected predictors for obesity at age 12.*

|  | 1 | 2 | 3 | 4 | 5 | 6 | 7 | 8 | 9 | 10 | 11 | 12 | 13 | 14 | 15 | 16 |
| --- | --- | --- | --- | --- | --- | --- | --- | --- | --- | --- | --- | --- | --- | --- | --- | --- |
| 1 | 1 |  |  |  |  |  |  |  |  |  |  |  |  |  |  |  |
| 2 | -0.04 |  |  |  |  |  |  |  |  |  |  |  |  |  |  |  |
| 3 | -0.16** | 0.12** | 1 |  |  |  |  |  |  |  |  |  |  |  |  |  |
| 4 | -0.29 | -0.01 | 0.42 | 1 |  |  |  |  |  |  |  |  |  |  |  |  |
| 5 | -0.02 | 0.22** | -0.03 | -0.10 | 1 |  |  |  |  |  |  |  |  |  |  |  |
| 6 | -0.06 | <0.01 | 0.06 | 0.02 | -0.05 | 1 |  |  |  |  |  |  |  |  |  |  |
| 7 | 0.35** | -0.17** | -0.54* | -0.40 | -0.02 | 0.03** | 1 |  |  |  |  |  |  |  |  |  |
| 8 | -0.29 | 0.18** | 0.38* | 0.31 | 0.12 | 0.17** | -0.46 | 1 |  |  |  |  |  |  |  |  |
| 9 | -0.35** | 0.06* | 0.46 | 0.81** | -0.07 | 0.12** | -0.47 | 0.41 | 1 |  |  |  |  |  |  |  |
| 10 | 0.25 | 0.13** | <0.01 | -0.03 | 0.23** | 0.10** | 0.08 | 0.02** | -0.03 | 1 |  |  |  |  |  |  |
| 11 | 0.28** | -0.15** | -0.43 | -0.39** | -0.14 | -0.08 | 0.29** | -0.28** | -0.37 | -0.04 | 1 |  |  |  |  |  |
| 12 | 0.19 | -0.10** | -0.25 | -0.29** | -0.12 | 0.04 | 0.31 | -0.19 | -0.27** | 0.09* | 0.37* | 1 |  |  |  |  |
| 13 | <-0.01 | -0.02 | 0.01 | -0.05 | -0.08 | <0.01 | 0.02 | <-0.01 | 0.01 | 0.02 | 0.03 | -0.12** | 1 |  |  |  |
| 14 | -0.05 | 0.20** | 0.06* | 0.08** | 0.05 | 0.03 | -0.06 | 0.10** | 0.08** | 0.06* | -0.05 | -0.09** | -0.04 | 1 |  |  |
| 15 | -0.21** | 0.08** | 0.29 | 0.42 | -0.07* | 0.09 | -0.37 | 0.27 | 0.42 | <0.01 | -0.23 | -0.23 | 0.08 | 0.05* | 1 |  |
| 16 | <0.01 | -0.02 | -0.09 | -0.06 | 0.14 | 0.02 | 0.04 | -0.02 | -0.06 | 0.01 | -0.02 | 0.05 | -0.21 | -0.02 | -0.12 | 1 |
| * p <.05; ** p <.01 | | | | | | | | | | | | | | | | |

1. Maternal age
2. Maternal BMI
3. Maternal education
4. Smoking in pregnancy
5. GDM/diabetes in pregnancy
6. Location
7. Household income
8. SIMD quintile
9. Indoors smoking in the household
10. Delivered by caesarean
11. Breastfeeding
12. Introduction of solid food
13. Child’s sex
14. Child’s BMI at age 5–6
15. ACEs count (5 categories)
16. PCEs count (3 categories)
